# Use of a Medial Femoral Condyle Free Flap for Metacarpal Reconstruction of Type IIIC Congenital Thumb Hypoplasia

**DOI:** 10.1016/j.jhsg.2026.101151

**Published:** 2026-09-25

**Authors:** Brooke E. Allen, Natalie Pawlak, Alec C. Smith, John A. Tipps, Evelyn R. Reed, Diala Burjak, Shaun D. Mendenhall

**Affiliations:** ∗ Division of Plastic and Reconstructive Surgery, Department of Surgery, Spencer Fox Eccles School of Medicine at the University of Utah, and Intermountain Primary Children’s Hospital, Salt Lake City, UT

**Keywords:** Congenital hand surgery, Congenital thumb hypoplasia, Congenital upper limb differences, Medial femoral condyle free flap, Metacarpal reconstruction

## Abstract

This is a case report of a 14-year-old girl with a Blauth Type IIIc hypoplastic thumb who underwent metacarpal reconstruction with a medial femoral condyle flap to preserve a five-digit hand. At the patient’s 9-month follow-up, imaging demonstrated complete bone healing at both the first metacarpal recipient site and the donor site. At 17 months after surgery, the patient is satisfied with the appearance and improved function as she now has a stable post with a small amount of thumb abduction and adduction, with minimal donor site morbidity. The patient has elected not to undergo the planned second stage of opposition transfer, first webspace deepening, and tendon transfer for the time being, although these procedures may increase her function further. This case presents a novel application of the MFC flap to preserve a five-digit hand in a patient with congenital thumb hypoplasia, with minimal donor site morbidity.

Thumb hypoplasia describes various congenital conditions that range from minor generalized thumb hypoplasia (Manske–Blauth Grade I) to thumb aplasia (Manske–Blauth Grade V).^1^ Thumb hypoplasia can markedly impair function because of the reliance on the thumb for the completion of complex motor tasks such as pinch and prehension. The primary reconstructive surgical technique for the management of Manske–Blauth Grade IIIB+ thumb hypoplasia is pollicization, in which the hypoplastic thumb is amputated and the index finger is repositioned as a thumb.^1^ Pollicization improves dexterity, but postoperative patients only retain four digits. Whether motivated by cultural values or personal preference, a family and a patient’s wish to preserve a five-digit hand is a valid consideration. Multiple reconstructive techniques have emerged to assist patients with Grade IIIB+ thumb hypoplasia who wish to preserve their thumb, such as toe phalanx transfer, hemimetatarsal transfer, or iliac crest transfer.^2^, ^3^, ^4^, ^5^ However, the study team is unaware of any literature that has described the use of the medial femoral condyle (MFC) flap for hypoplastic thumb reconstruction. Given the graft’s flexibility and vascularity, the MFC flap is a versatile reconstructive technique that boasts low donor site morbidity.^6^ This case report describes a novel metacarpal reconstructive technique with a vascularized osteocutaneous MFC flap to preserve a five-digit hand in a 14-year-old girl with a Blauth Type IIIc hypoplastic thumb.

## Case Report

### Preoperative management

A 14-year-old girl with left-sided Blauth Type IIIc thumb hypoplasia presented to our pediatric hand surgery clinic for thumb reconstruction (Figure 1, Figure 2, Figure 3). Both the patient and her family desired to improve her hypoplastic thumb’s function (Video S1, available online on the Journal’s website at https://www.jhsgo.org), but they wanted to preserve her five-digit hand. Preoperative imaging demonstrated Type IIIc thumb hypoplasia, with a mostly absent first metacarpal and a congenital absence of the radial artery (Figure 4, Figure 5). In addition to her thumb hypoplasia, the patient had a past medical history of radioulnar synostosis, and VACTERL association. The patient had an extensive previous surgical history for management of her lower gastrointestinal and genitourinary congenital differences, but she had not had any previous upper-extremity surgeries. She had no baseline medication requirements.

**Figure 1:**
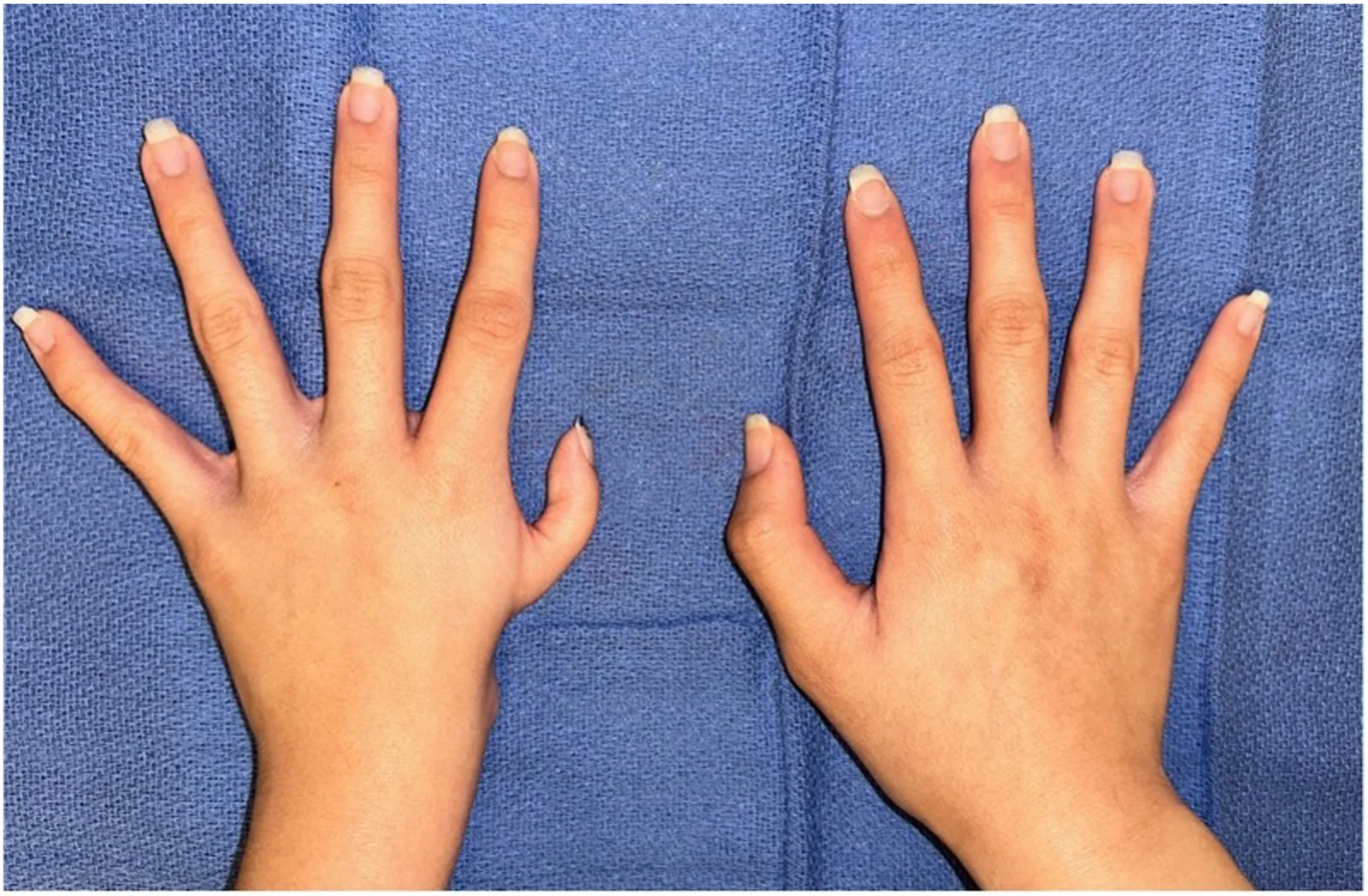
Preoperative dorsal view of an adolescent girl with left-sided, Type IIIc thumb hypoplasia. The patient had no history of previous upper-extremity surgery but has VACTERL association and radioulnar synostosis. Printed with permission from and copyrights retained by Shaun D. Mendenhall, MD.

**Figure 2:**
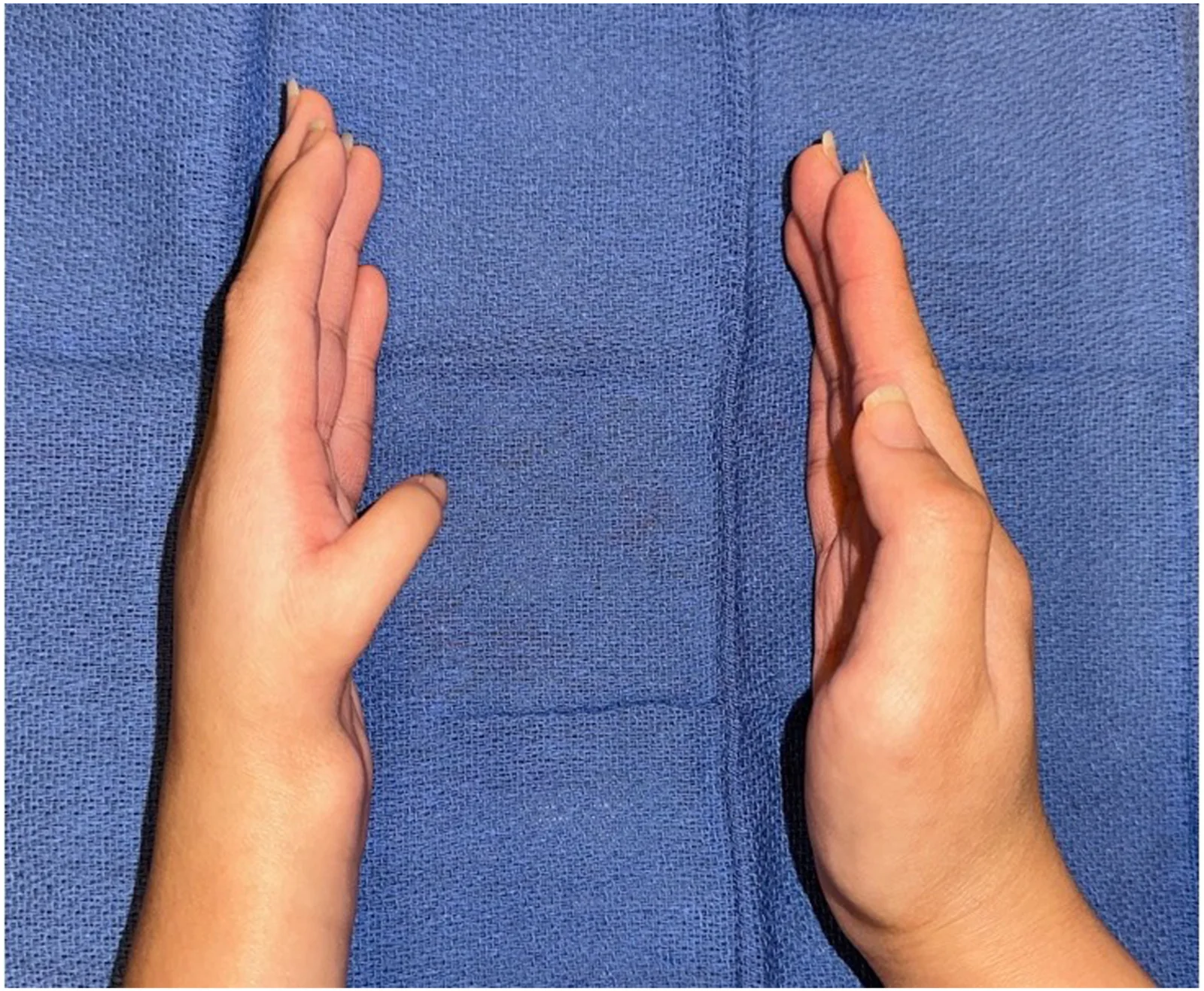
Radial (lateral) view of the patient’s hands prior to left first metacarpal reconstruction. Printed with permission from and copyrights retained by Shaun D. Mendenhall, MD.

**Figure 3:**
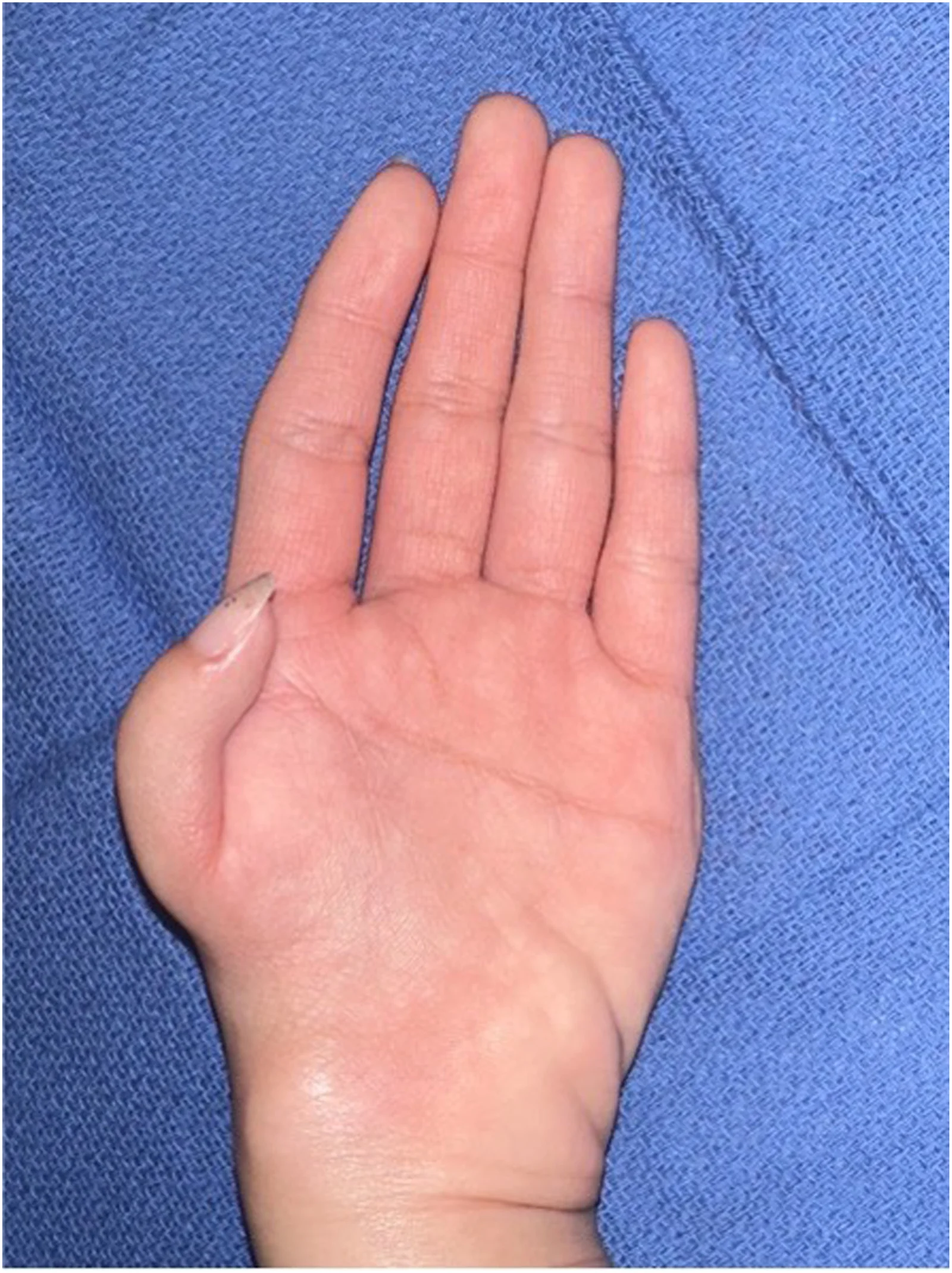
Palmar view of the patient’s left hand prior to first metacarpal reconstruction with a MFC osteocutaneous flap. Printed with permission from and copyrights retained by Shaun D. Mendenhall, MD.

**Figure 4:**
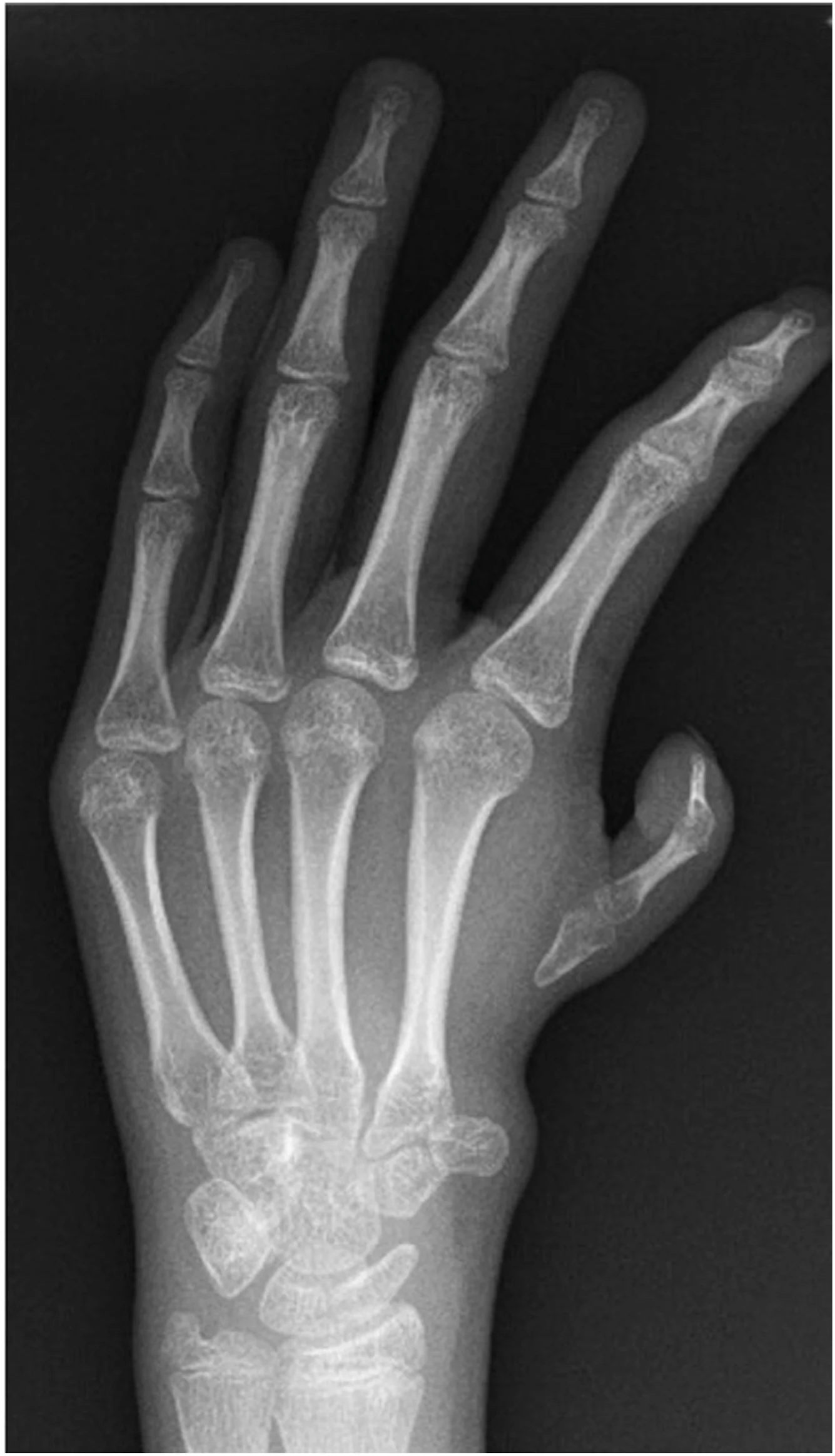
Preoperative imaging of the patient’s left-sided Type IIIc thumb hypoplasia, which demonstrates a rudimentary first metacarpal. Printed with permission from and copyrights retained by Shaun D. Mendenhall, MD.

**Figure 5:**
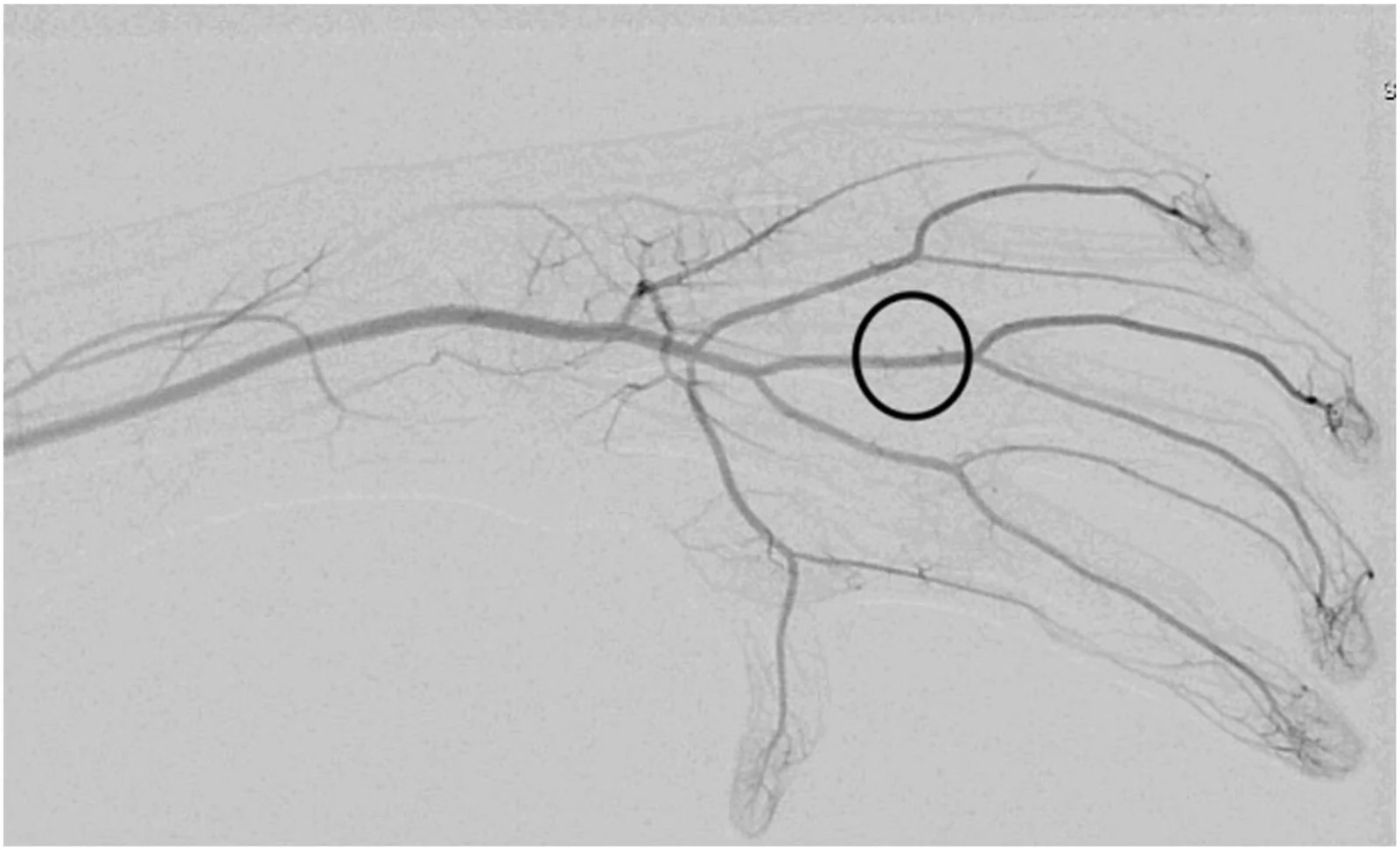
Preoperative angiogram demonstrating aberrant vascular anatomy with absence of the radial artery and a dominant ulnar artery. The circle marks the site of the recipient vessel for the MFC flap, which was the common digital artery to the third webspace. Printed with permission from and copyrights retained by Shaun D. Mendenhall, MD.

During the discussion of surgical alternatives to pollicization, other reconstructive techniques were considered less favorable than the MFC flap. The MFC flap was selected over the iliac crest graft because of the ease of flap raising and lower donor site morbidity. In addition, toe phalanx or metatarsal transfers have higher donor site morbidity than the MFC flap and the potential for aesthetic alterations to the foot such as toe shortening or floppiness.^6^, ^7^, ^8^ Despite there being surgical techniques like a split metatarsal transfer to the excised metatarsal to avoid toe shortening, the surgical team elected to mitigate any risks of morbidity or aesthetic changes to the foot.^9^ Moreover, the vascularized MFC flap’s skin paddle capabilities for thenar eminence reconstruction are advantageous over nonvascularized techniques.

### Intraoperative management

The patient was prepared with both an upper and lower-extremity tourniquet in place. An intraoperative Doppler was used prior to making incisions, with audible Doppler signals in the wrist and palm marked as potential recipient vessels. The upper-extremity tourniquet was inflated, and an initial incision was made directly over where the Doppler signal was found at the wrist. The exploration confirmed the congenital radial artery’s absence, and a small anterior interosseus artery that was unsuitable as a recipient vessel. Furthermore, the ulnar artery and its branches were too far away to use without a vein graft. A separate incision was made in the palm, where the common digital artery and a small vein to the third webspace were isolated and found to be suitable in size for anastomosis (Fig. 6). The flap recipient site over the hypoplastic metacarpal was then incised, and the proximal spike of the metacarpal neck remnant was located. Using a rongeur, the spike was removed to create a distal docking point for the bone flap without disrupting the metacarpophalangeal collateral ligaments. Two 0.9 mm (0.035 in) K-wires were then directed in an antegrade fashion through the metacarpal head, into the base of the proximal phalanx, and of the distal thumb tip in preparation for a bony fixation of the MFC flap. Finally, the distal aspect of the trapezium was identified and decorticated in preparation for insetting the proximal MFC bone flap (Fig. 7).

**Figure 6:**
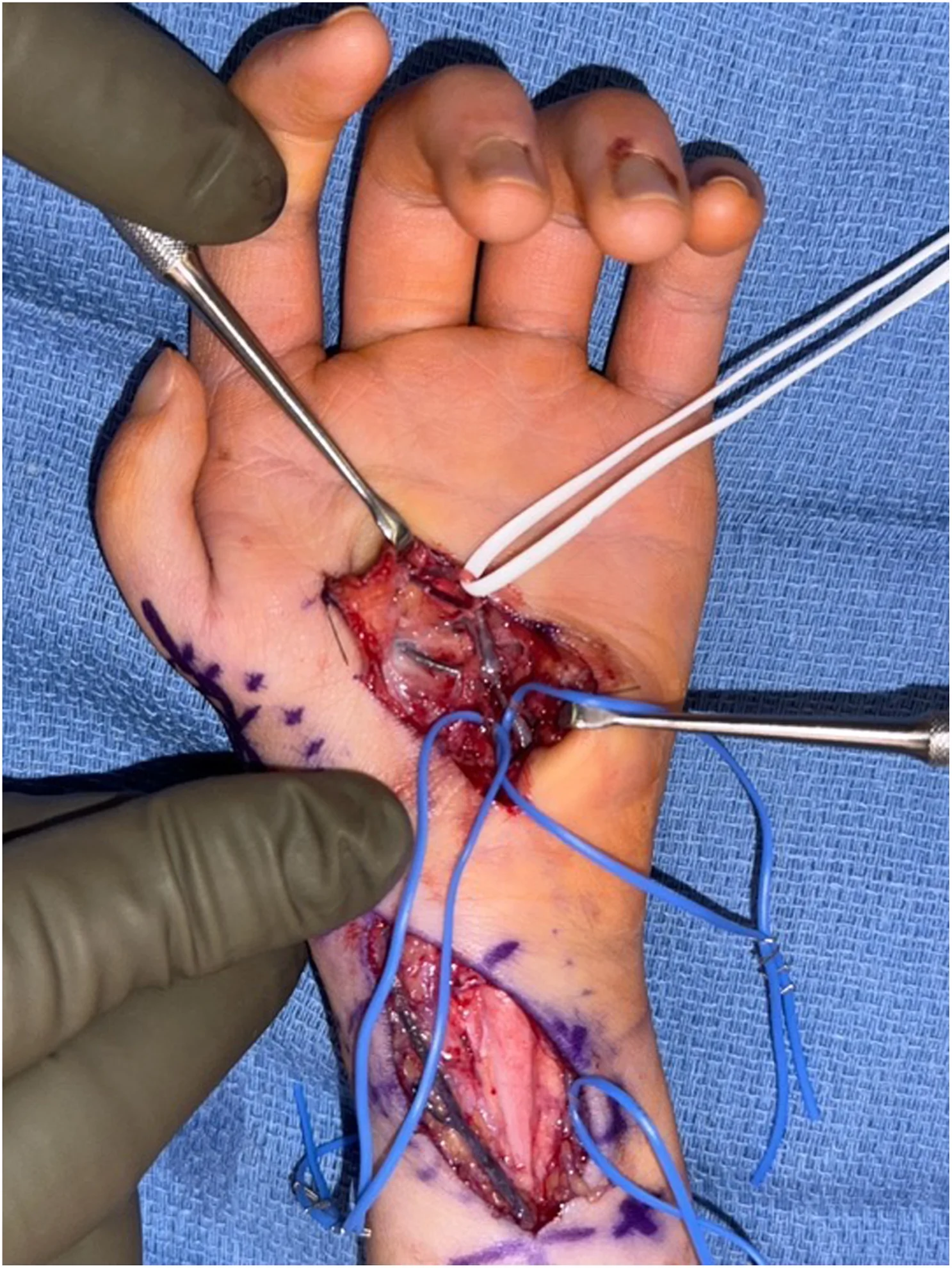
A Doppler was used before surgery to identify potential recipient vessel sites within the palm and wrist. The initial incision was made along the wrist but neither the ulnar artery nor the anterior interosseus artery were suitable recipient vessels. A second incision was then made in the palm. The common digital artery (white) and a vein (blue) to the third webspace were isolated as recipient vessels. Printed with permission from and copyrights retained by Shaun D. Mendenhall, MD.

**Figure 7:**
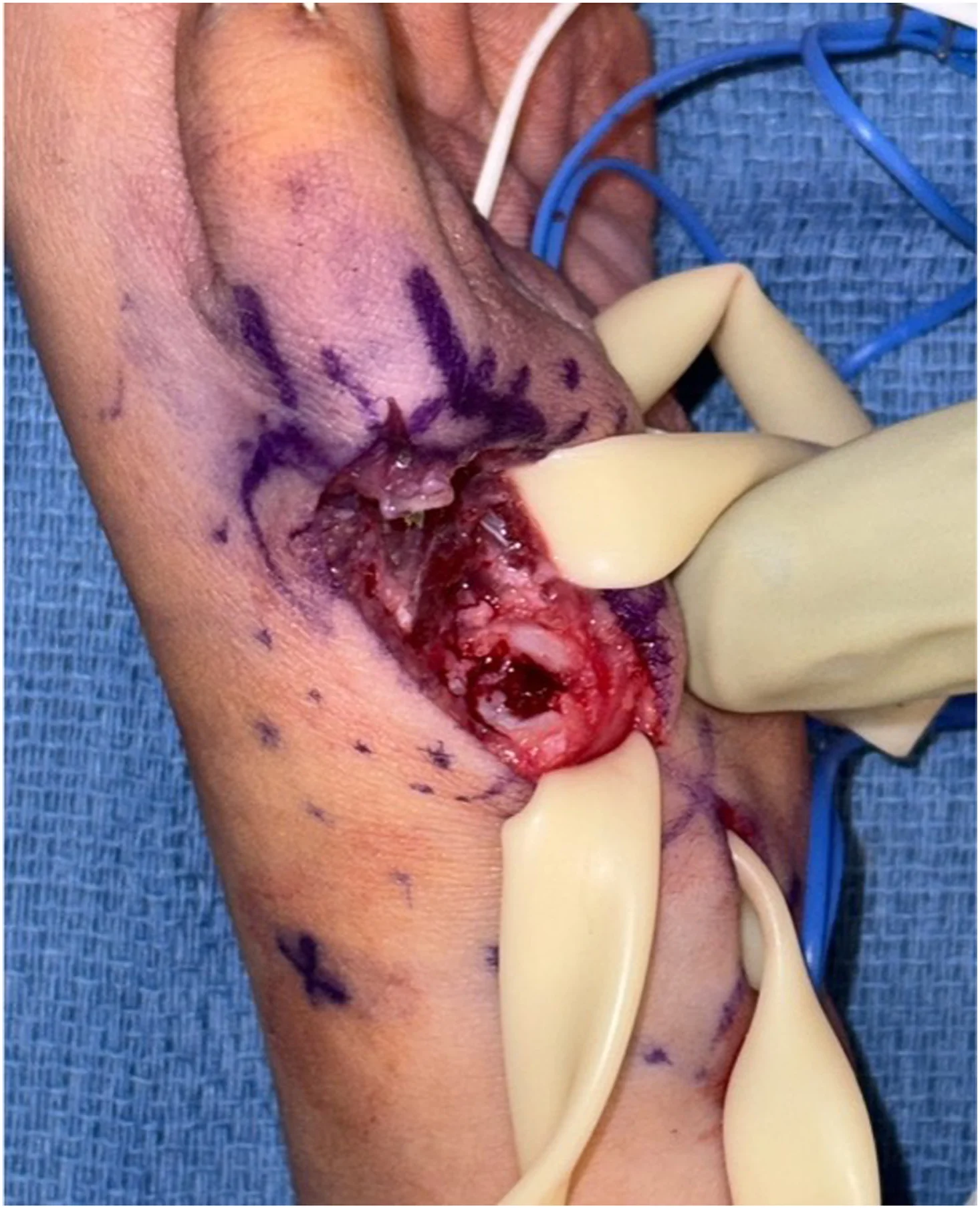
Bone preparation with osteotomy at the metacarpal neck to remove the rudimentary spike of bone, antegrade K-wire placement, and decortication of the trapezium. Printed with permission from and copyrights retained by Shaun D. Mendenhall, MD.

The osteocutaneous MFC flap was raised in a standard fashion under tourniquet.^7^ The descending genicular artery pedicle was found just posterior to the vastus medialis muscle. A microsagittal saw was then used to cut the bone graft, which measured as 1.5 cm wide by 2.5 cm long. An osteotome was used to excavate the bone. Flap inset was performed under fluoroscopy, and the previously placed K-wires were drilled retrograde from the metacarpal head through the MFC graft and into the native trapezium to achieve bony fixation. The bone flap was inset with the thumb in a slightly palmar abducted position to correct for the patient’s adduction deformity. Microanastomoses of the artery and single vein were then performed to the common digital vasculature going to the third webspace. Because of excessive bulk and the inability to inset the skin paddle, the skin and subcutaneous tissue were debulked, leaving the deep fascia covering the bone flap. The defatted skin was then used as a full thickness skin graft (Fig. 8).

**Figure 8:**
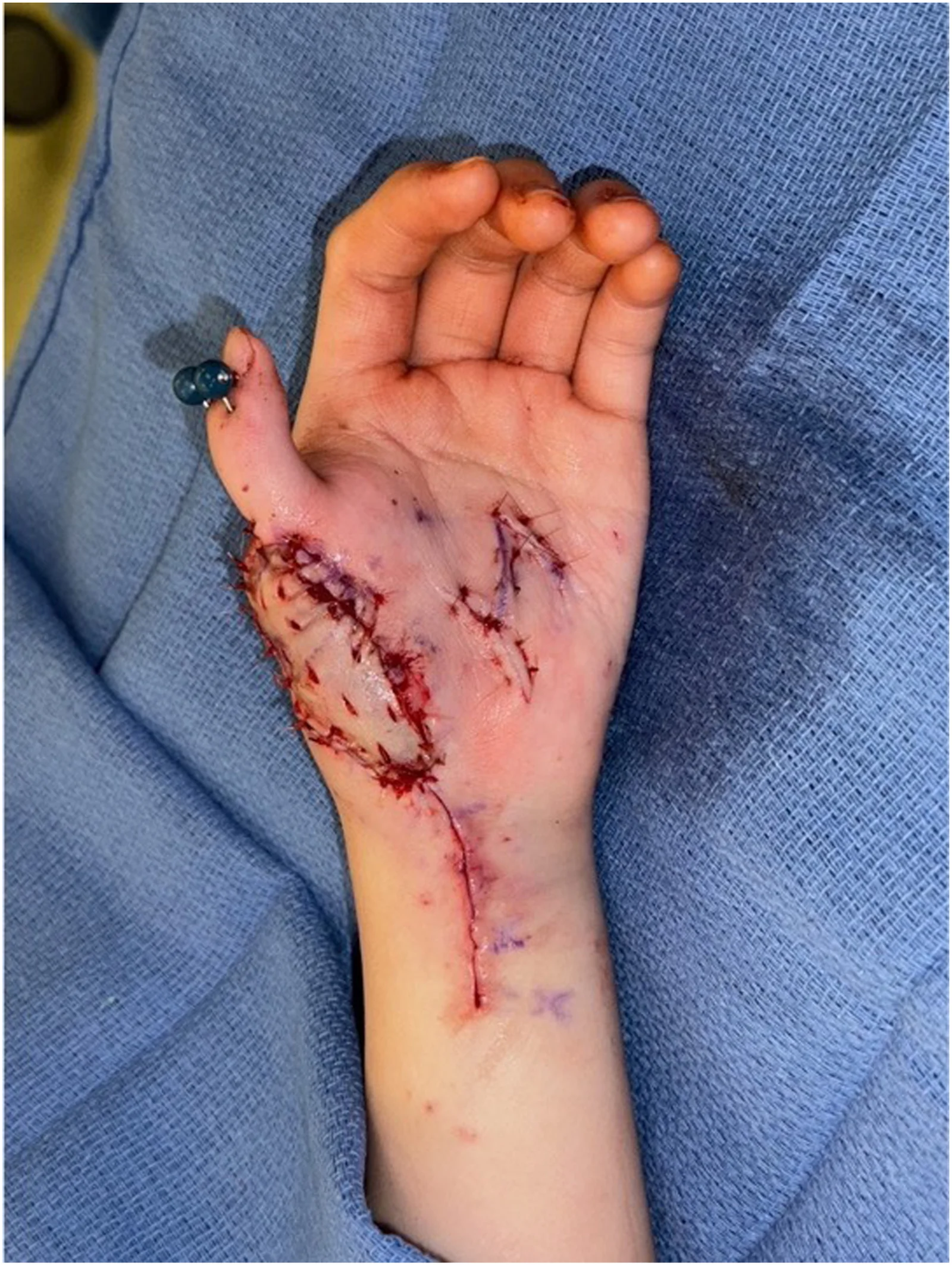
Immediate postoperative view of the hand after first metacarpal reconstruction with an osteo-fascial MFC flap with a full thickness skin graft. Printed with permission from and copyrights retained by Shaun D. Mendenhall, MD.

### Postoperative management

Patient was admitted to the pediatric intensive care unit for observation and neurovascular checks every 4 hours. Postoperative anticoagulation consisted of 81 mg aspirin for 30 days. She was discharged on postoperative day 1. Four days after surgery, dressing changes demonstrated well-healing incision sites and decent early skin graft take. The patient was able to bear weight on and flex the donor knee without significant pain. At 7 weeks after surgery, the patient did not endorse pain in either her donor or flap recipient site, and her pins were removed. She had slight movement at the carpometacarpal joint and was cleared for hand therapy. Her thenar eminence had improved both in volume and appearance. Her sensation was intact to the thumb tip apart from diminished sensation at her flap site. At her nine-month follow-up, imaging demonstrated complete bone healing at both the donor and flap sites (Figure 9, Figure 10, Figure 11). During her physical examination, patient was able to pinch to the side of her index finger, but she had no isolated movement of her thumb interphalangeal joint. Her carpometacarpal joint demonstrated limited flexion and extension and her metacarpophalangeal joint was relatively stable. The patient and her caregivers were overall happy with the increased stability of her thumb (Table S1, available online on the Journal’s website at https://www.jhsgo.org), with their only concerns being the widening of her donor site scar as well as the hyperpigmentation of both surgical sites (Fig. 12). To optimize her range of motion and function, the surgical team originally planned a Huber opposition transfer, first webspace deepening, and possible extrinsic tendon transfer as the next stage in her reconstruction. However, the patient and her family have decided to postpone further surgery at this time, as they are happy with the appearance and functional improvement of her thumb (Figure 13, Figure 14, Figure 15). The patient and her legal guardian provided written consent to publish this case.

**Figure 9:**
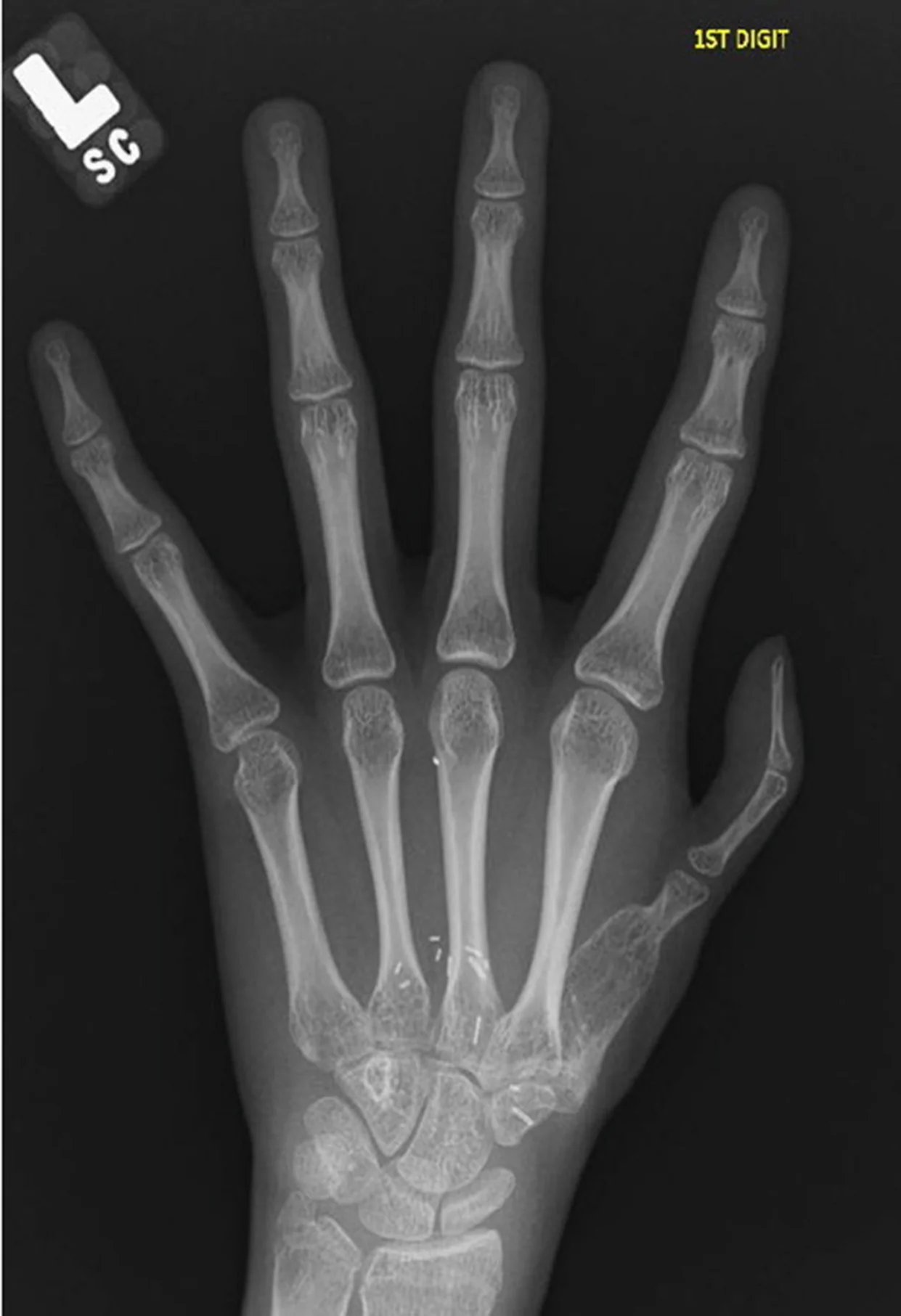
PA view x-ray of left thumb at 9 months after surgery. Flap recipient site demonstrates complete bony healing. Printed with permission from and copyrights retained by Shaun D. Mendenhall, MD.

**Figure 10:**
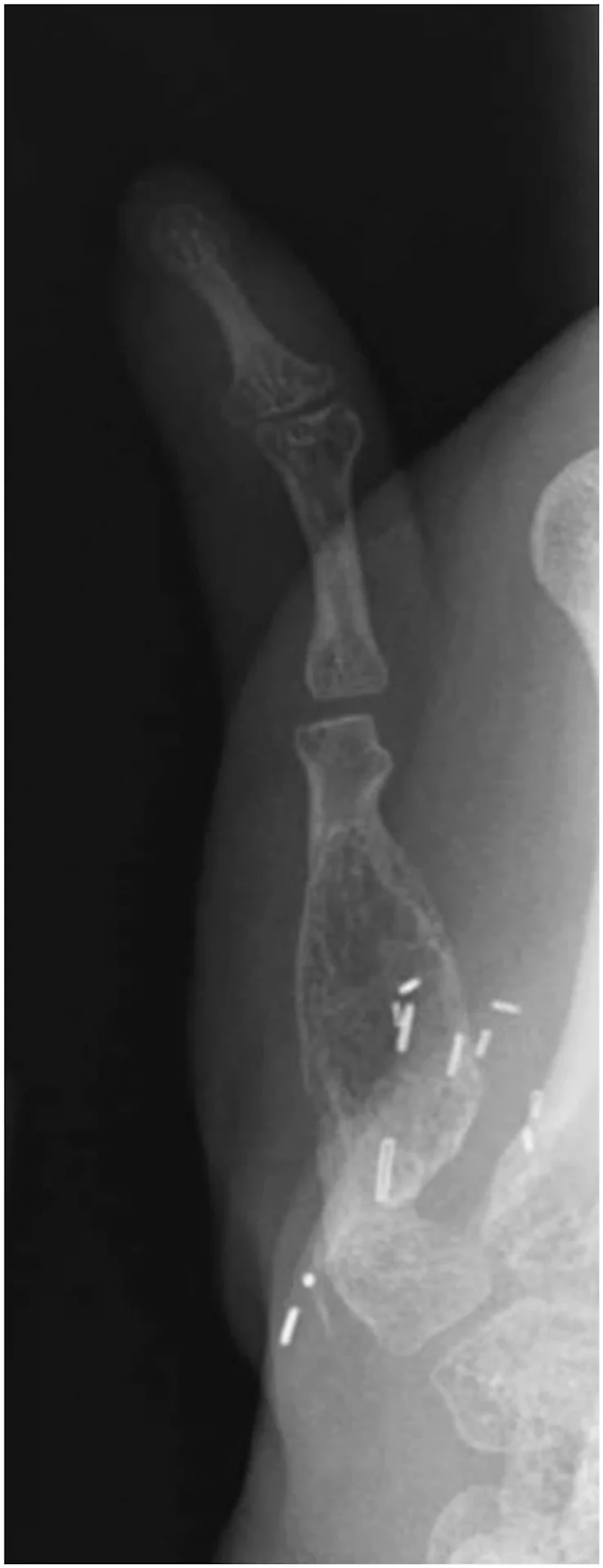
Oblique view x-ray of the left thumb at 9 months after surgery, with complete bony healing at the flap inset. Printed with permission from and copyrights retained by Shaun D. Mendenhall, MD.

**Figure 11:**
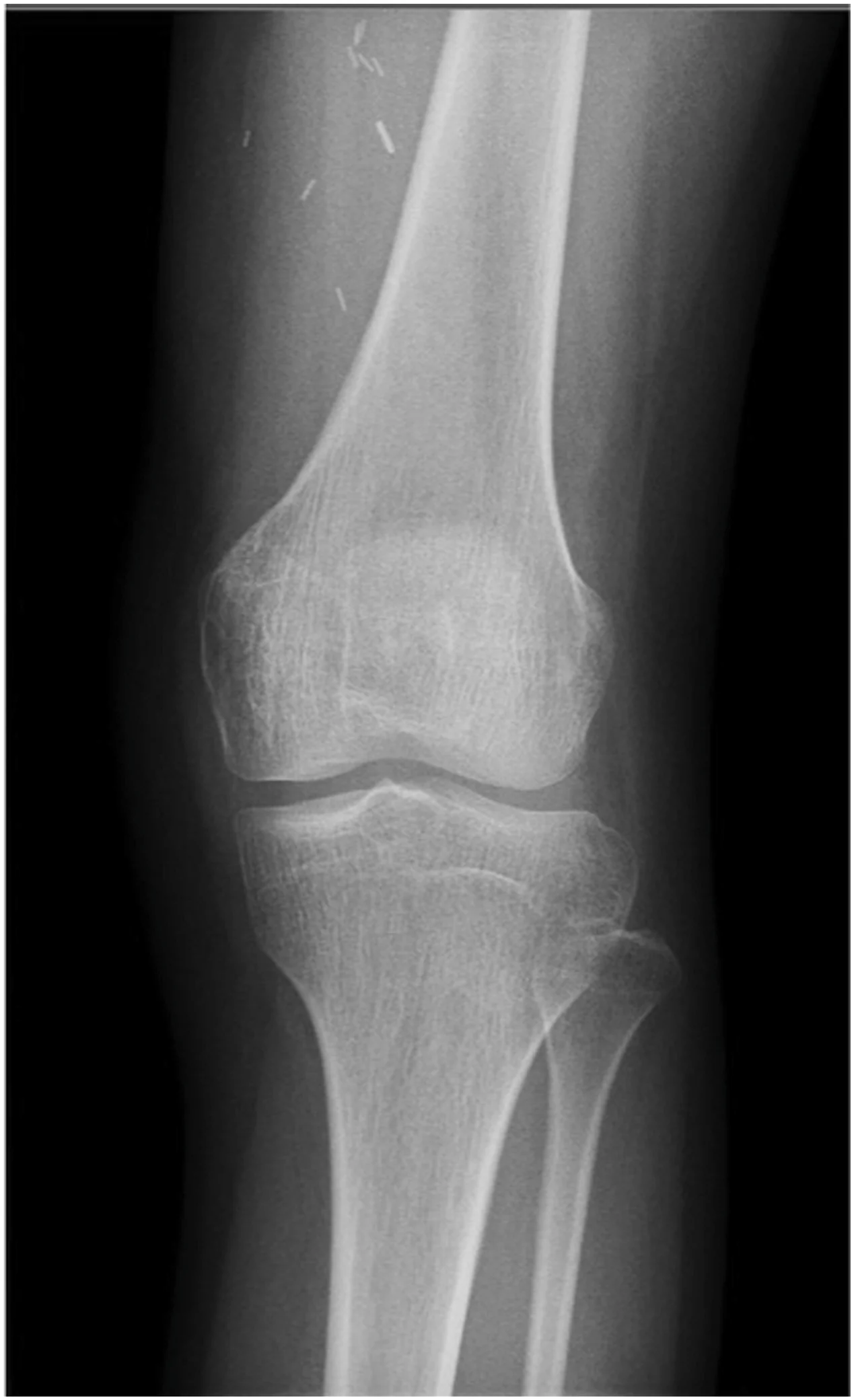
An x-ray of a flap donor site within the left knee at 9 months after surgery showing healing of the defect. Printed with permission from and copyrights retained by Shaun D. Mendenhall, MD.

**Figure 12:**
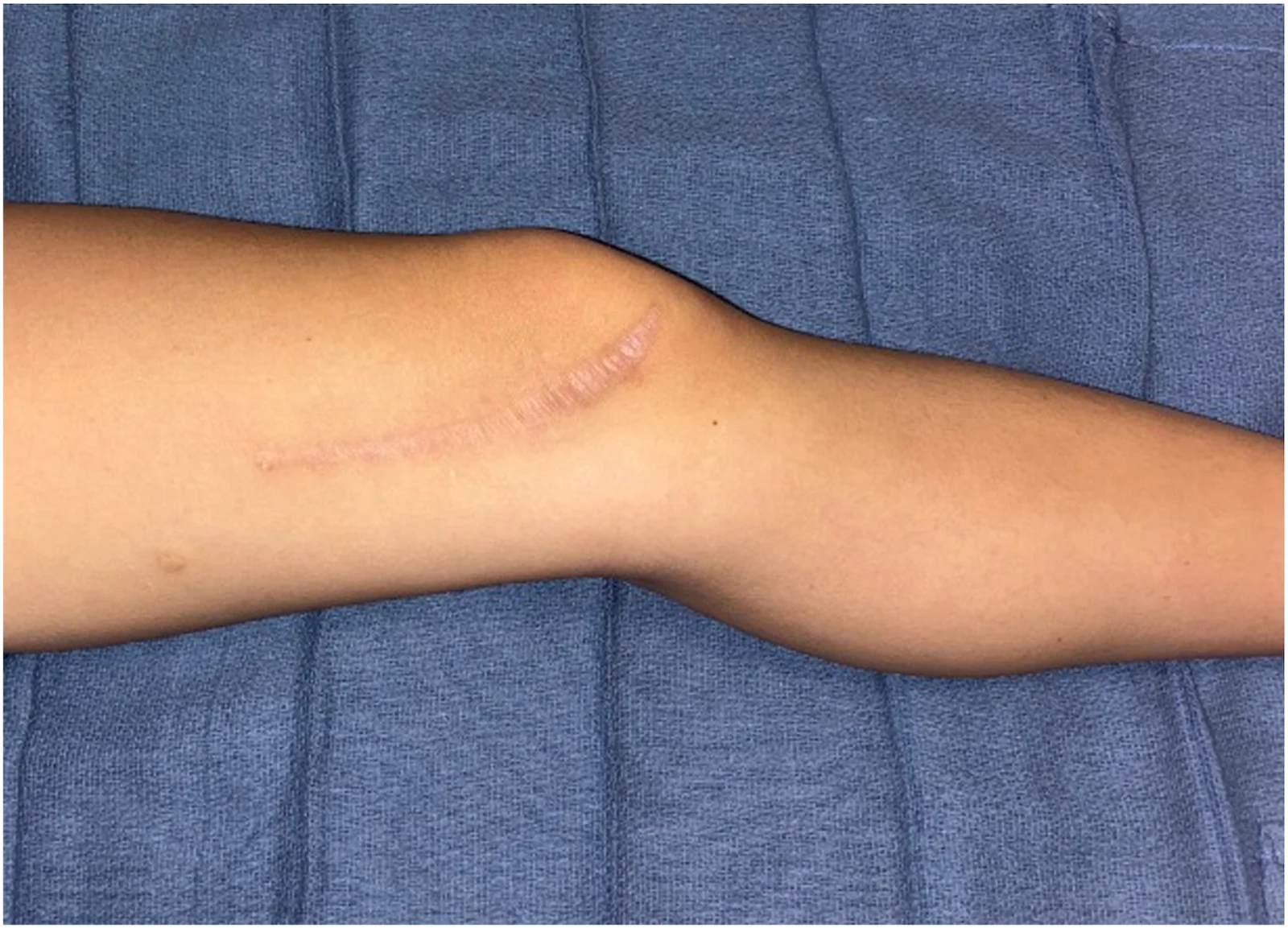
Knee donor site scar at 16 months after surgery. Printed with permission from and copyrights retained by Shaun D. Mendenhall, MD.

**Figure 13:**
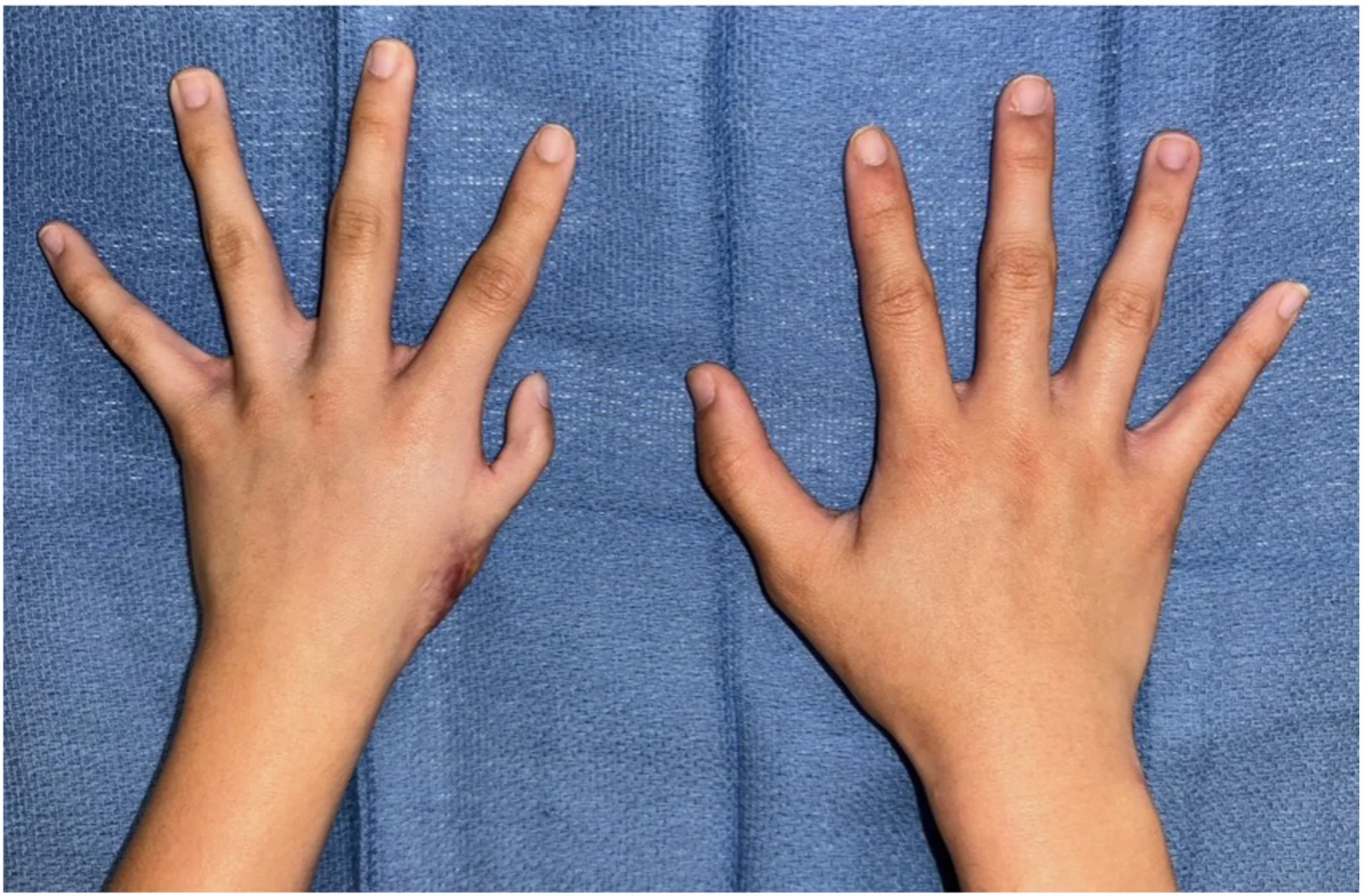
Dorsal view of the hands at 9 months after surgery. Printed with permission from and copyrights retained by Shaun D. Mendenhall, MD.

**Figure 14:**
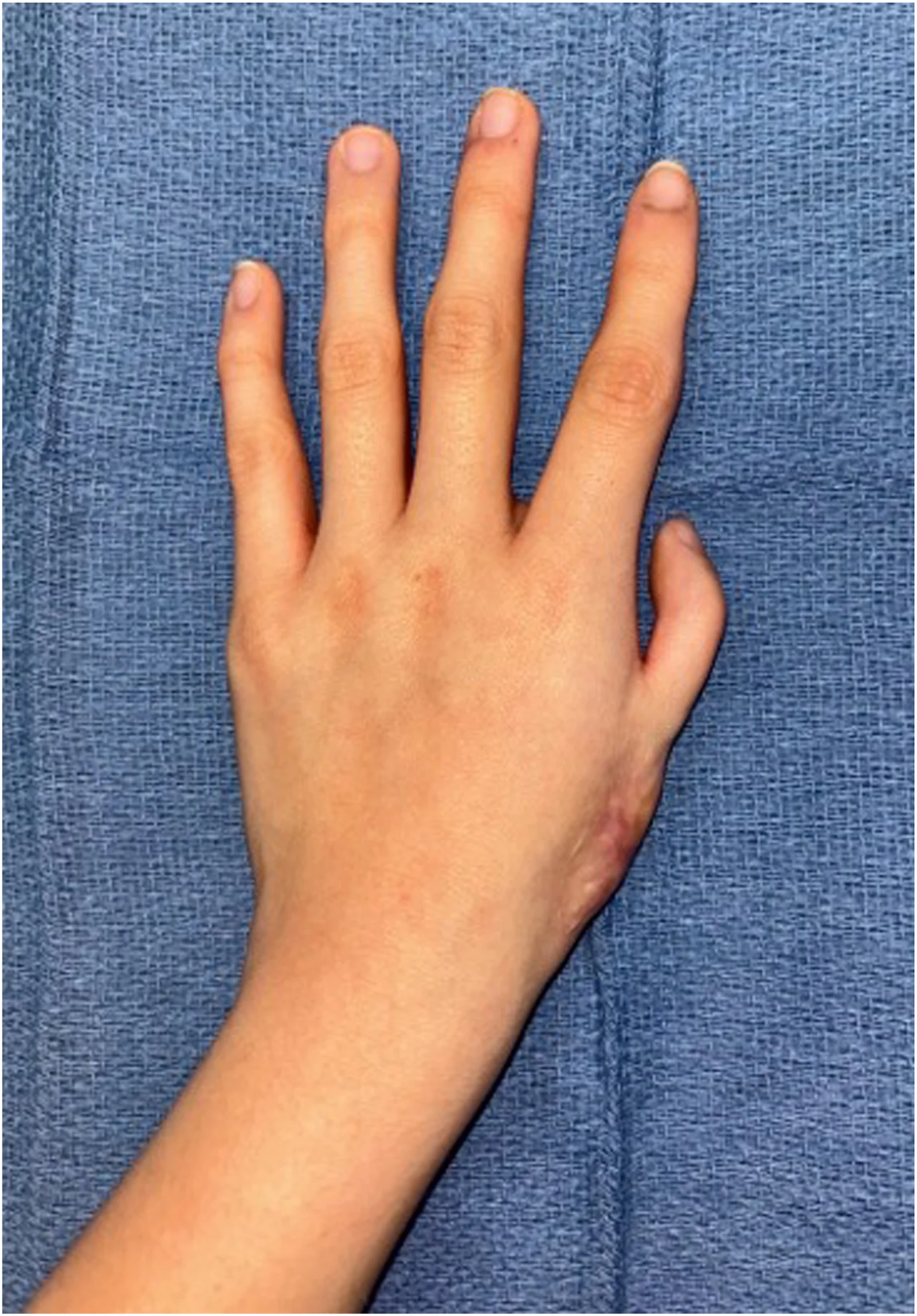
Dorsal view of the hand at 16 months after surgery. Printed with permission from and copyrights retained by Shaun D. Mendenhall, MD.

**Figure 15:**
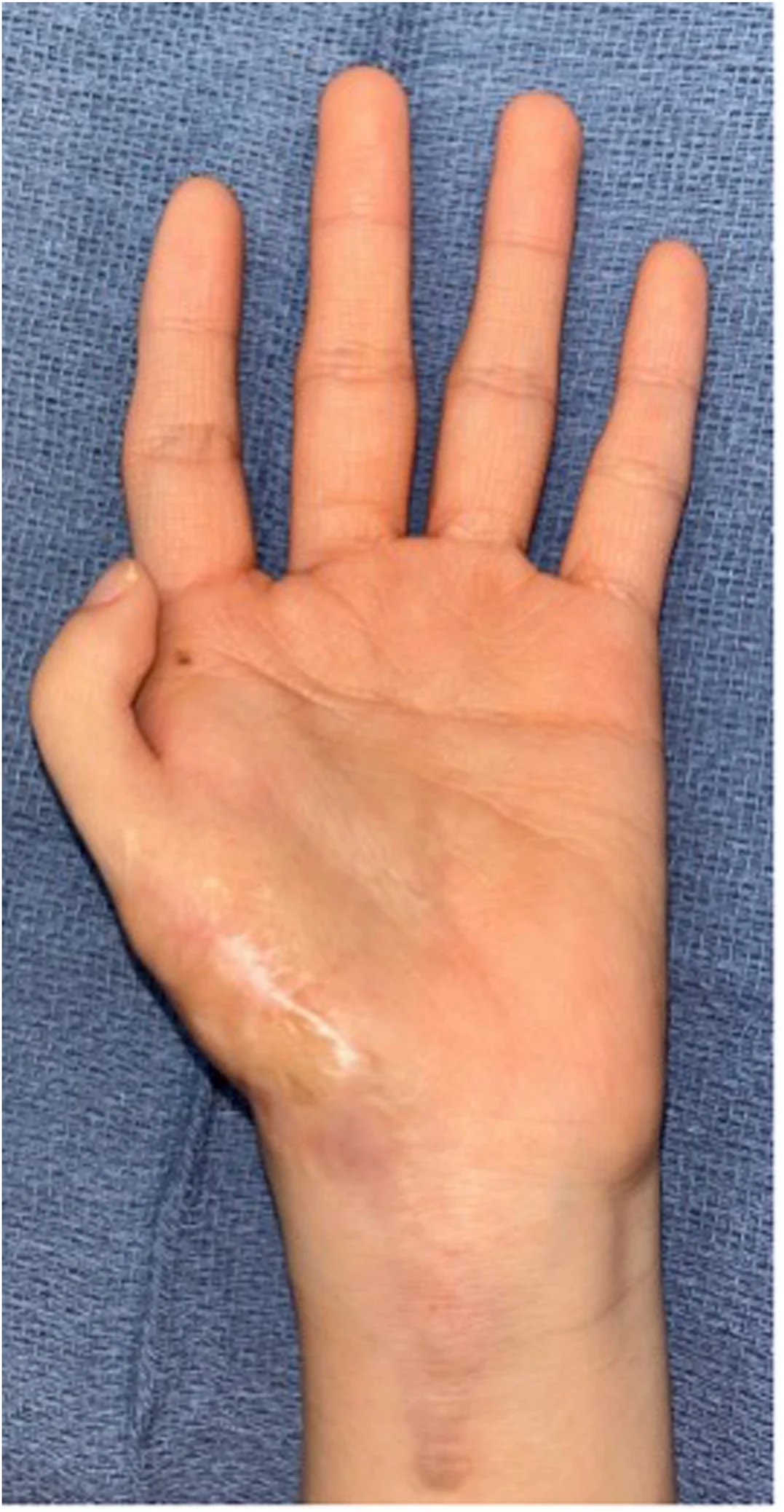
Volar view of the hand at 16 months after surgery. Printed with permission from and copyrights retained by Shaun D. Mendenhall, MD.

## Discussion

To the best of our knowledge, this is the first reported case using the MFC flap for metacarpal reconstruction of a Blauth Type IIIc hypoplastic thumb.^5^ The MFC flap has become a key component of our practice for reconstructing problematic nonunions and small bone defects. This is because of its straightforward dissection, robust vascularity, and ability to include muscle or skin paddles for composite reconstruction.^6^^,^^7^ In this case, the flap helped us achieve our goals of creating a more stable thumb with a better appearance albeit with the addition of scarring. The donor site of the MFC flap is quite favorable in comparison to other donor sites as it does not alter major arterial blood supply to the limb, allows immediate ambulation, and has a low complication risk.^6^^,^^7^

Although pollicization would undoubtedly lead to superior measurable functional outcomes, this patient’s desire for a five-digit hand superseded these functional gains. As surgeons, our primary motivation is often to optimize function, but the patient’s sense of wholeness and associated improved social functioning should be of equal importance when selecting a reconstructive technique. Taking this into consideration, preservation of the hypoplastic thumb using a MFC flap was the appropriate technique in this case. The best next step for our patient to improve functional outcomes will be to perform the second stage procedures noted previously.

Reconstruction of a hypoplastic with an MFC flap is not without limitations. Given the relatively small diameter of the descending geniculate artery of 2.1 mm in adults, the vessel’s small size in young children may prove to be technically challenging for many surgeons.^10^ Furthermore, avoiding the distal femoral physis is an additional challenge for using MFC flaps in skeletally immature children. Considering these factors, it would be difficult to perform a MFC flap for hypoplastic thumb reconstruction in younger children, making nonvascularized options more reasonable.^5^^,^^8^

### Conclusion

The MFC osteocutaneous flap is a suitable reconstructive technique in older children for Blauth Type IIIB+ thumb hypoplasia. This alternative allows for the maintenance of a five-digit hand, which may be preferable to patients and their families who prioritize a sense of wholeness. This case demonstrated favorable outcomes in a 14-year-old patient with Blauth Type IIIc thumb hypoplasia.

## Ethics declaration

Written informed consent to take part in the study and to publish the article has been obtained from all participants or their legal representatives. The privacy rights of participants have been observed.

This study was performed in compliance with relevant laws, regulatory frameworks and guidelines where the research took place. Ethics committee approval was not required under relevant laws and institutional guidelines. Single case reports do not require an IRB at our institution.

This research follows the CARE guidelines and the CARE checklist.

## Consent to Publish

Written informed consent to publish this study and images was provided by the participant’s legal guardian.

## Conflicts of Interest

SDM is an educational consultant for PolyNovo. No benefits in any form have been received or will be received by the other authors related directly to this article.

## Supplementary Data

Video 1 Video demonstrating the thumb’s range of motion in the before surgery and after surgery (17 months) periods. Used with permission from and copyrights retained by [REDACTED].
